# Glyoxalase-I Is Upregulated in Acute Cerulein-Induced Pancreatitis: A New Mechanism in Pancreatic Inflammation?

**DOI:** 10.3390/antiox10101574

**Published:** 2021-10-05

**Authors:** Marcus Hollenbach, Sebastian Sonnenberg, Ines Sommerer, Jana Lorenz, Albrecht Hoffmeister

**Affiliations:** Medical Department II—Oncology, Gastroenterology, Hepatology, Pneumonology, Infectious Diseases, Division of Gastroenterology, University of Leipzig Medical Center, Liebigstrasse 20, D-04103 Leipzig, Germany; ines.sommerer@medizin.uni-leipzig.de (I.S.); jana.lorenz@medizin.uni-leipzig.de (J.L.); albrecht.hoffmeister@medizin.uni-leipzig.de (A.H.)

**Keywords:** glyoxalase-I, AR42J, cerulein, dexamethasone, siRNA, overexpression, amylase

## Abstract

Inflammation caused by oxidative stress (ROS) demonstrates an essential mechanism in the pathogenesis of acute pancreatitis (AP). Important sources for ROS comprise the reactive compound methylglyoxal (MGO) itself and the MGO-derived formation of advanced glycation end-products (AGEs). AGEs bind to the transmembrane receptor RAGE and activate NF-κB, and lead to the production of pro-inflammatory cytokines. MGO is detoxified by glyoxalase-I (Glo-I). The importance of Glo-I was shown in different models of inflammation and carcinogenesis. Nevertheless, the role of Glo-I and MGO in AP has not been evaluated so far. This study analyzed Glo-I in cerulein-(CN)-induced AP and determined the effects of Glo-I knockdown, overexpression and pharmacological modulation. Methods: AP was induced in C57BL6/J mice by i.p. injection of CN. Glo-I was analyzed in explanted pancreata by Western Blot, qRT-PCR and immunohistochemistry. AR42J cells were differentiated by dexamethasone and stimulated with 100 nM of CN. Cells were simultaneously treated with ethyl pyruvate (EP) or S-p-bromobenzylglutathione-cyclopentyl-diester (BrBz), two Glo-I modulators. Knockdown and overexpression of Glo-I was achieved by transient transfection with Glo-I siRNA and pEGFP-N1-Glo-I-Vector. Amylase secretion, TNF-α production (ELISA) and expression of Glo-I, RAGE and NF-κB were measured. Results: Glo-I was significantly upregulated on protein and mRNA levels in CN-treated mice and AR42J cells. Dexamethasone-induced differentiation of AR42J cells increased the expression of Glo-I and RAGE. Treatment of AR42J cells with CN and EP or BrBz resulted in a significant reduction of CN-induced amylase secretion, NF-κB, RAGE and TNF-α. Overexpression of Glo-I led to a significant reduction of CN-induced amylase levels, NF-κB expression and TNF-α, whereas Glo-I knockdown revealed only slight alterations. Measurements of specific Glo-I activity and MGO levels indicated a complex regulation in the model of CN-induced AP. Conclusion: Glo-I is overexpressed in a model of CN-induced AP. Pharmacological modulation and overexpression of Glo-I reduced amylase secretion and the release of pro-inflammatory cytokines in AP in vitro. Targeting Glo-I in AP seems to be an interesting approach for future in vivo studies of AP.

## 1. Introduction

Acute pancreatitis (AP) is one of the most common gastrointestinal admission diagnoses worldwide. Despite improvements in interventional therapeutic approaches, AP is still associated with a significant morbidity and mortality as well as a considerable socioeconomic burden [1]. Genetic mutations, bile duct stones, alcohol consumption and others lead to an imbalance of pancreatic proteases and antiproteases. As a consequence, a premature intrapancreatic protease activation and consecutive autodigestion lead to cell death and inflammation involving damage-associated molecular patterns (DAMPs) and the nucleotide-binding oligomerization domain-like receptor protein 3 (NLRP3) inflammasome pathway [2,3,4]. Finally, several mechanisms result in the stimulation of the transcription factor NF-κB with production of pro-inflammatory cytokines and oxidative stress (ROS). Thus, the infiltration of immune cells to the disordered pancreas contributes to the severity of pancreatitis and leads to a vicious cycle of disease [5]. Moreover, studies indicated that ischemia and reperfusion are additional important mechanisms for the development or aggravation of AP and are also associated with ROS [6]. In this regard, AP can be, amongst others, a result of cardiac [7] or vascular [8] surgery. In addition, experimental studies show that ischemia alone may initiate the development of AP and could aggravate pancreatic damage, whereas the improvement of pancreatic blood flow reduces the severity of AP [6,9,10].

On molecular levels, ROS can originate from so-called “dicarbonyl stress”. “Dicarbonyl stress” is characterized by a α-oxoaldehyde-induced glycation of protein residues (mainly arginine) that leads to mitochondrial protein dysfunction, enzyme inactivation, mutagenesis, apoptosis and immune response [11,12]. “Dicarbonyl stress” is mainly caused by methylglyoxal (MGO) that is formed as a by-product in glycolysis [13], ketone body metabolism and threonine catabolism [14,15,16]. The highly reactive MGO modifies nucleotides, phospholipids and proteins [17,18], that finally result in a rapid formation of so-called advanced glycation end-products (AGE). In addition, reducing sugars such as glucose react with amino groups and trigger MGO formation as well as AGE generation non-enzymatically within the Maillard reaction [19]. AGEs also induce several pro-inflammatory cellular processes, production of ROS and activate different signaling pathways via the transmembrane receptor for advanced glycation end-products (RAGE). The activation of RAGE stimulates the phosphorylation of numerous signaling pathways (e.g., the extracellular signal regulated kinase 1/2 (ERK1/2), phosphoinositide 3-kinase (PI3-K)/protein kinase B (AKT), Janus kinase 2 (JAK2), TGF-β, vascular epithelial growth factor (VEGF) and RhoGTPases) that active NF-κB and result in the production and release of pro-inflammatory cytokines [11].

To protect cells from the MGO-induced detrimental effects, MGO is detoxified by the glyoxalase system. Thereby, glyoxalase-I (Glo-I) catalyzes the conversion of α-oxo-aldehydes such as MGO and L-glutathione (GSH) to form the corresponding hemithioacethal, S-D-lactoylglutathione [20]. Consecutively, glyoxalase-II (Glo-II) converts S-D-lactoylglutathione to D-lactate and restores GSH. Herein, Glo-I is the rate-limiting enzyme in this series of reactions [21]. Thereby, (partial) Glo-I inhibitors (e.g., ethyl pyruvate (EP) or S-p-bromobenzylglutathione cyclopentyl diester (BrBz)), that modulate the specific enzymatic activity, revealed protective effects in models of inflammation or carcinogenesis [11,22,23,24,25,26].

Despite the involvement of Glo-I in several inflammatory processes and the induction of cancer [22,23,24,25], the role of Glo-I in AP has not been investigated so far. In this study, (I) we analyzed Glo-I in the model of cerulein (CN)-induced pancreatitis in vivo, (II) examined the effect of a pharmacological modulation of Glo-I on a cell culture model of AP and (III) studied the consequences of Glo-I overexpression and knockdown. Thus, our study underlines the potential role of Glo-I for future studies on AP.

## 2. Materials and Methods

### 2.1. Animal Experiments

C57BL6/J mice aged 10 to 12 weeks (16; 8 per group, 4 female and 4 male animals in each group) were used for animal experiments. All animal experiments were approved by the local ethical review board (animal research proposal TVV 50/11; Ethical Committee at the Medical Faculty, Leipzig University; IORG0001320, IRB00001750, chairwoman: Prof. Dr. med. Dr. phil. Ortrun Riha, Käthe-Kollwitz-Str. 82, D-04109 Leipzig). All regional rules and regulations of the European Union (guideline 86/609/EWG) and the local animal protection authorities were followed. Animals were kept under pathogen-free conditions in type-II cages (four animals per cage, separated by gender) with dry sluice. Mice were kept at 22 ± 2 °C on a 12 h light/dark cycle with electronic admission control, an automated air conditioner and filtering. Standard local hygienic management was used according to “Hygiene Monitoring of Mice and Rats in Various Housing Systems”, recommended by GV-SOLAS. All mice had free access to water at all times, were fed a standard chow (4% energy from fat, Altromin Spezialfutter, Lage, Germany) and were weighed every day. Mice were observed for typical symptoms, e.g., alterations of body weight, coat, behavior (apathy) and food intake, to calculate a score by GV-SOLAS. The experiments were stopped if the score reached 20 or more points, followed by euthanasia with cervical dislocation.

In mice, an AP can be induced as mild or severe form depending on number and days of CN injections [27]. In our study, AP was induced using an adapted mouse model of CN-induced mild acute pancreatitis, as previously described [28,29,30,31,32]. Prior to injection of CN or saline, mice were fasted for 14–16 h with free access to water. AP was induced by ten hourly intraperitoneal (i.p.) injections of CN (50 μg/kg body weight, Bachem, Bubendorf, Switzerland) followed by cervical dislocation one hour after the last injection. Control animals received 0.9% sterile saline (Sigma-Aldrich, Steinheim, Germany) at the same time points. Collection of blood samples was performed under isofluran (Baxter, Deerfield, IL, USA) sedation at baseline (prior to first injection) and after 11 h of treatment. Amylase and glucose measurements of animal sera were performed via ELISA by the Institute of Laboratory Medicine of the University of Leipzig Medical Center (see below).

### 2.2. Cell Culture and Cerulein Treatment

Rat pancreatic AR42J acinar cells were purchased from ATCC (CRL1492, Manassas, VA, USA) and maintained in DMEM (high glucose, Biochrom/Merck, Berlin, Germany), supplemented with 10% fetal calf serum (FCS, Biochrom/Merck) and 1% penicillin/streptomycin (P/S, PAA, Pasching, Austria). AR42J cells derived from Wistar rats are adherent cells with secretory activity following induction with glucocorticoids [33]. Cells were kept at standardized conditions (37 °C with 5% CO_2_) and medium was replaced every 48 h. AR42J cells were passaged once a week. Cells were detached with trypsin (Biochrom/Merck). If cells were thawed from frozen stocks, they were initially supplemented with DMEM and 40% FCS. After thawing, cells were allowed to grow and acclimate for 4 to 6 weeks (4 to 6 passages) prior to performing experiments [33].

For experiments, cells were placed in 6-well plates (TPP, Sigma-Aldrich) at concentrations of 6 × 10^5^ cells/well (for transfection) or 2 × 10^6^ cells/well (for all other experiments). Medium without FCS was used. Dexamethasone (dexa, Sigma-Aldrich) was added at concentrations of 100 nM in a final volume of 2 mL. Cells were necessarily incubated for 48 h to allow differentiation to an acinar-like phenotype (dexa pretreatment). Medium was changed after 24 h.

After dexa pretreatment, experiments were performed. Medium was changed and 100 nM of dexa with or without 100 nM of CN ((Bachem), final volume per well 2 mL) was added in serum-free medium for 1 up to 24 h. Controls received dexa only. Supernatants were collected for amylase secretion assays. For some experiments, CN and/or the Glo-I enzyme modulators EP (1–20 mM) or BrBz (1–10 µM, both Sigma-Aldrich) [22,23,24] were added. RNA isolation was performed using the RNeasy Mini Kit (74104, Qiagen, Hilden, Germany) following the instructions of the manufacturer. For protein isolation, a protein lysis buffer (RIPA with complete ultra-tablets protease inhibitor high complete, Roche, Mannheim, Germany) was added to the cells. Collected samples were stored at −80 °C.

### 2.3. Immunohistochemistry

After cervical dislocation of animals, the pancreata were immediately removed and fixed in formaldehyde solution (4%, OttoFischerGmbH, Saarbruecken, Germany) for at least 24 h, embedded in paraffin and cut into 5 µm thick longitudinal sections. One section was mounted on one slide. Quantitative analysis included six slides per pancreas (each tenth), that were equally distributed over the organ. Sections were dewaxed and rehydrated by xylol (Sigma-Aldrich) following a descending ethanol (Sigma-Aldrich) series. Subsequently, slides were boiled in target retrieval solution (pH 9, Dako, Hamburg, Germany) for 30 min and cooled down for 15 min. Then, sections were washed thrice in PBS (Biochrom/Merck) with 0.025% tween 20 (PBST, Sigma-Aldrich). Quenching of endogenous peroxidase activity with 3% H_2_O_2_ (Sigma-Aldrich) was performed thrice (2 × 10 min, 1 × 5 min), followed by another washing step. Slides were blocked with 5% donkey serum (host of secondary antibody, Sigma-Aldrich) for 30 min at room temperature and incubated with primary antibodies (AB) against Glo-I (human, anti-mouse, monoclonal, SC-133214, Santa-Cruz, TX, USA) or RAGE (human, anti-mouse, monoclonal, SC-365154, Santa-Cruz) for 60 min at room temperature. Slides were then washed thrice and incubated with secondary AB (715-035-150, donkey, anti-mouse polyclonal AB, Dianova, Hamburg, Germany) for 30 min at room temperature. Staining was performed with Diaminobenzidin (DAB, Peroxidase Substrate Kit (Liquid DAB + Substrate Chromogen System, Dako)), following the instructions of the manufacturer. Slides lacking the primary antibody were used as controls. Sections were counterstained with ready-to-use hematoxylin (Hollborn, Leipzig, Germany). Supplemental overview images and images for quantification of pancreatic damage were stained with hematoxylin (Hollborn) and eosin (0.2%, Medite, Burgdorf, Germany; HE). Stained sections were captured, and stacked images were compiled with a Biozero BZ 8000 microscope (Keyence, Osaka, Japan). Staining intensity was calculated according to the Quick Score (Q): results were scored by multiplying the percentage of positive cells by the intensity (Q = P × I; maximum = 300). Intensity was determined as 1+, 2+ or 3+ according to absent, partial or complete staining [34].

The histologic examination of pancreatic damage was quantified as described before [35]. Six slides per pancreas were analyzed for grading of edema (0 = no, 1 = interlobular, 2 = + moderate intralobular, 3 = + severe intralobular edema), leukocytic infiltration (0 = absent, 1 = scarce perivascular, 2 = moderate perivascular and scarce diffuse, 3 = abundant diffuse infiltration), vacuolization (0 = absent, 1 = <25%, 2 = 25–50%, 3 = >50% of acinar cells), hemorrhagia (0 = no, 1 = 1–2, 2 = 3–5, 3 = >5 hemorrhagic foci per slide) and necrosis (0 = no, 1 = <15%, 2 = 15–35%, 3 = >35% of cells).

For staining of AR42J cells, 50,000 cells were dropped on a cytospin (Thermo-Fisher Scientific, Waltham, MA, USA) and centrifuged for 3 min at 500 rpm. Cells were fixed in 4% formaldehyde (OttoFischerGmbH) solution for 20 min at room temperature. Spins were washed thrice in PBS (Biochrom/Merck) and permeabilized with 0.1% Triton-X 100 (Sigma-Aldrich) for 5 min at room temperature. After washing with 0.025% PBST (Biochrom/Merck, Sigma-Aldrich, see above), quenching of endogenous peroxidase activity by 3% H_2_O_2_ (Sigma-Aldrich) for 10 min was performed twice, followed by another washing step. The corresponding primary antibodies (see above) were incubated overnight at 4 °C and secondary antibodies (see above) for 60 min at room temperature.

### 2.4. qRT-PCR

RNA was isolated by means of the RNeasy Mini Kit (Qiagen) according to the instructions in the manual. For quantitative real-time PCR (qRT-PCR), the QuantiTect SYBR Green RT PCR Kit (one-step PCR, Qiagen 204243) was used following the instructions of the manufacturer. The following QuantiTect Primer Assays (Qiagen) were used: Glo-I (QT00438984), HPRT (QT00199640), β-actin (QT00193473), GAPDH (QT00199633), RPL7 (QT02524774) and PPIB (QT00169736). PPIB was used as a housekeeping gene for experiments as it was not influenced by CN (Supplementary Figure S4). Experiments were performed on a Light Cycler 3.5 (Roche), and results were calculated as mean of at least 8 animals per group by the Relative Expression Software Tool (REST^®^, Qiagen).

### 2.5. Western Blot Analysis

Protein lysates were broiled for 5 min at 95 °C in SDS protein buffer (5 × laemmli sample buffer, Thermo Fisher Scientific) and separated by sodium dodecyl sulfate polyacrylamide gel electrophoresis (SDS-PAGE) following transfer to a polyvinylidene fluoride (PVDF) membrane (both Roth, Karlsruhe, Germany). Primary antibodies against Glo-I (SC-133214), NF-ĸB (p65 subunit, mouse, anti-mouse, -rat, -human, monoclonal SC-8008, Santa-Cruz), RAGE (SC-365154), β-Actin (mouse, anti-mouse, -rat, -human and other, C-15, A5441, Sigma-Aldrich), PPIB (anti-cyclophilin-B, rabbit, anti-mouse, polyclonal, ab16054, abcam, Cambridge, United Kingdom) and Vinculin (mouse, anti-human, -mouse, -rat, -avian, monoclonal, SC-73614, Santa-Cruz) were used. Vinculin was used as a housekeeping marker for Western blot as it was not influenced by CN (Supplementary Figure S4). Secondary antibodies were anti-mouse (goat, anti-mouse, polyclonal 1858413) and anti-rabbit (goat, anti-rabbit, 1858415, polyclonal, both Pierce/Thermo Fisher Scientific). Western blot lanes were quantified using an imager (G-Box Chemie XX9, Syngene, Cambridge, UK). Signals were normalized to their respective loading controls using ImageJ Software (v. 1.48, http://imagej.nih.gov, accessed on 13 August 2021) and GeneTools (Syngene, Frederick, ML, USA)

### 2.6. Measurement of Specific Glo-I Activity

Activity of Glo-I (E.C.4.4.1.5) was determined by measurement of the reaction intermediate S-D-lactoylglutathione, with ascending absorbance at 240 nm. Absorbance was measured for 5 min at 25 °C in a high-throughput microplate assay (Greiner UV-star 96-well plate, Frickenhausen, Germany) with a photometer (Infinite M200, Tecan, Maennedorf, Switzerland). For each test, 2 mM of GSH (Sigma-Aldrich) and 2 mM of MGO (Roth) were incubated for 45 s in 50 mM of phosphate buffer (Na_2_HPO_4_, pH 7.0, Roth), and 4 µL of undiluted cell lysate was used per test (200 µL final volume per well). Each probe was measured three times. Phosphate buffer (Sigma-Aldrich) was set as a reference. Enzyme activity was calculated in U by the formula: A = (ΔE/min × V)/(ε × d × v). ε for S-D-lactoylglutathione was 2.86 (mol/L × cm). For specific activity, U refers to protein concentration [36].

### 2.7. Measurement of Amylase Secretion, TNF-α and MGO by ELISA

Supernatants of 6-well plates were thawed and measured via ELISA by the Institute of Laboratory Medicine of the University of Leipzig Medical Center. The AMYL2 Cobas^®^ kit (Roche) was used with adherence to the manufacturer’s instructions. Results refer to protein concentrations. International SI units were used (µkat/L = U/L × 0.0167). Measurements were performed at Cobas 8000 c502 (Roche). Frozen protein lysates were thawed on ice. For TNF-α-ELISA (BD Rat TNF ELISA, 560479, Becton Dickinson, NJ, USA) and MGO-ELISA (Rat Methylglyoxal ELISA kit, MBS2605842, MyBioSource, Vancouver, Canada) measurements, 20 µL of supernatant or protein lysate (24 h of treatment, as indicated) was used according to the instructions of the manufacturer. Results refer to protein concentrations.

### 2.8. Glo-I Plasmid Generation

Total RNA was isolated from AR42J cells using the RNeasy Mini Kit (Qiagen) following the manual. First-strand cDNA was generated from normalized RNA amounts using Oligo(dT)-primers and the Omnisript RT Kit (205111, Qiagen) according to the instructions of the manufacturer. Glo-I insert with Bgl-II and Eco-RI cutting sites was constructed with Glo-I primers (forward: GACAGATCTATGGCAGAGCCACAGCCA, reverse: CAGGAATTCCTAAATAATTGTTGCCATTTTGTT). The insert and a pEGFP-N1 vector (6085-1, Clontech, CA, USA) were digested by Bgl-II (R0144S, NEB, MA, USA) and EcoRI (R0101S, NEB). Dephosphorylation, ligation, transformation and midi preparation were performed as described before [37].

### 2.9. Transfection

Cells were seeded in 6-well plates (TPP) at concentrations of 6 × 10^5^ cells/well in serum-free medium containing 100 nM of dexa. Cells were incubated for 48 h with a medium change after 24 h. For transfection, 2.5 µg of plasmid DNA (pEGFP-N1-Glo-I or pEGFP-N1 control) and transfection reagent (Lipofectamin2000, Invitrogen, Thermo Fisher Scientific) were used following the instructions of the manufacturer. The 6-well plates were incubated on a shaker (50 rpm, Thermo Fisher Scientific) for 20 min at 37 °C with 5% CO_2_. Then, well plates were incubated for 24 h at 37 °C and 5% CO_2_.

For siRNA transfection, Lipofectamin RNAi Max (13778-150, Thermo Fisher Scientific), 30 pmol Glo-I-siRNA (Silencer Pre. Designed siRNA Rat Glyoxalase 1, 201921, Thermo Fisher Scientific) or control-siRNA (Silencer negative control 1 siRNA, AM4611, Applied Biosystems/Thermo Fisher Scientific) were used. Subsequent to transfection for 24 h, CN treatment was performed as described above.

### 2.10. Statistical Analysis

Results are expressed as mean ± standard deviation (SD). For comparison of only two groups, the Student’s test or Mann–Whitney U-test were performed. For three or more groups, the one-way ANOVA with Bonferroni post-test was used. *p*-values < 0.05 were considered statistically significant. All experiments represent means of at least three independent experiments. GraphPad Prism 4.0 software (San Diego, CA, USA) was used for the calculation and drawing of graphs.

## 3. Results

### 3.1. Glo-I Is Upregulated in CN-Induced AP in Mice

The induction of AP by CN was assured by histologic evaluation of pancreatic sections. Sections were stained with hematoxylin and eosin and indicated a unique increase in edema and leukocyte infiltration. In contrast, we did not register necrosis and only a slight increase in vacuolization and hemorrhage, confirming the induction of mild pancreatitis (Supplementary Figure S1C,D). In addition, CN-treated mice revealed mean amylase values of 154.3 ± 47.5 µkat/L, compared to 55.6 ± 11.5 µkat/L of the control animals (*p* < 0.001, Supplementary Figure S1A). Glucose levels of CN-treated animals decreased to 5.7 ± 2.1 vs. 8.9 ± 1.1 mmol/L (*p* = 0.002, Supplementary Figure S1B).

**Figure 1:**
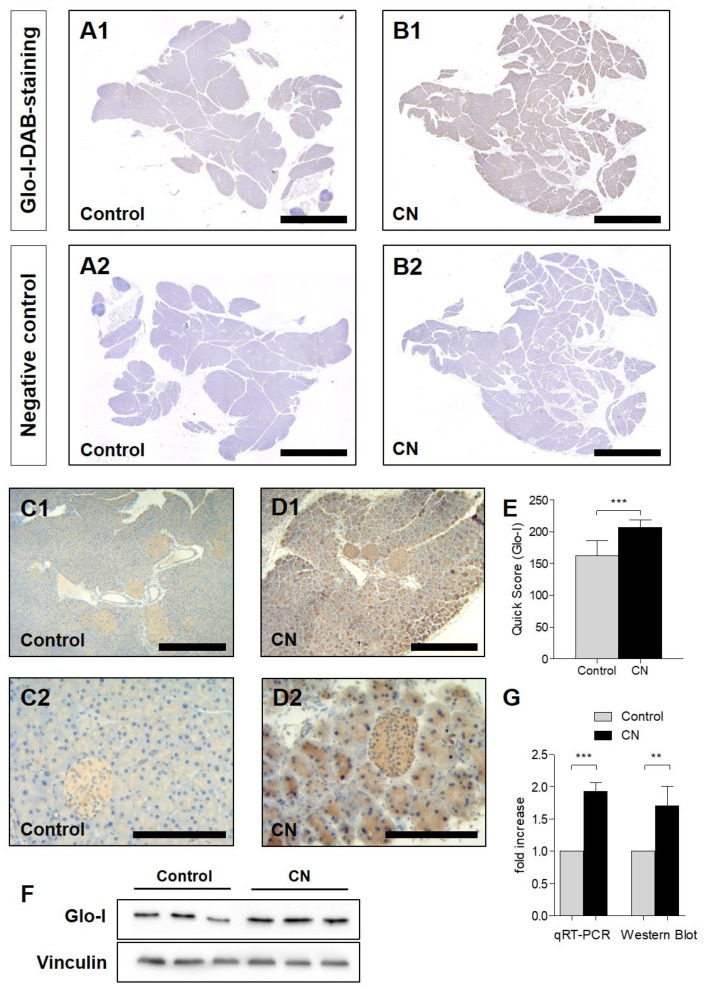
Glo-I in control and CN-treated mouse pancreata. (**A1**–**B2**) Representative Glo-I-DAB-stained bright-field images of stacked whole pancreata. CN treatment resulted in increased staining intensity (**A1**,**B1**). Staining controls lacking the primary antibody confirm specific Glo-I staining (**A2**,**B2**). Glo-I-DAB staining at 5× (**C1**,**D1**) and 20× magnification (**C2**,**D2**) shows a ubiquitous cytosolic Glo-I protein expression in pancreatic parenchyma and high expression in islets. Quantification by Quick Score confirms a statistically significant increase in Glo-I staining intensity in CN-treated mice (**E**). This upregulation was also found on mRNA levels by qRT-PCR and protein levels on Western blot, showing a 1.7–1.9-fold increase (**G**). (**F**) Representative Western blot images of three animals treated with CN or controls. Results are expressed as mean ± SD from 8 animals (4 male, 4 female) per group. Scale bars: 1000 µm (**A1**–**B2**), 100 µm (**C1**–**D2**). *** *p* < 0.001. Glo-I: Glyoxalase-I, CN: cerulein, DAB: Diaminobenzidin.

Glo-I-DAB-staining of explanted pancreata clearly indicated an elevated Glo-I protein expression in CN-treated animals. Stacked overview images (Figure 1(A1–B2)) and higher magnifications (C1–D2) show a ubiquitous cytosolic Glo-I expression with higher staining intensity in AP animals (B1,D1,D2). Furthermore, pancreatic islets also revealed a high staining intensity of Glo-I. Quantifications by means of the Quick Score demonstrated a mean score of 162.0 ± 24.3 for controls, compared to 207.1 ± 11.5 for CN-treated mice (*p* < 0.001, Figure 1E). Results of Western blot or qRT-PCR also showed a significant increase of Glo-I expression in mice with CN-induced AP (Figure 1F,G).

### 3.2. Glo-I and RAGE Expression in AR42J Cells

**Figure 2:**
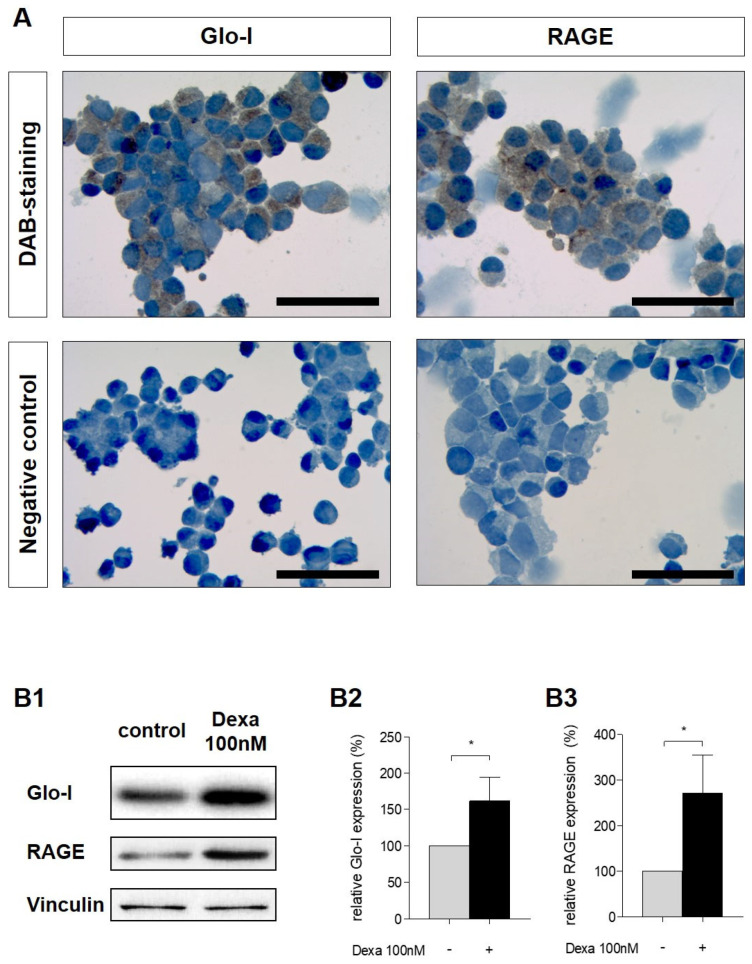
Expression of Glo-I and RAGE in AR42J and effect of dexamethasone. (**A**) Representative Glo-I- and RAGE-DAB-staining images of AR42J cells stained using cytospin. Negative controls lack the primary antibodies. Images indicate ubiquitous cytosolic Glo-I protein expression and predominant membrane RAGE protein expression. (**B1**–**B3**) effect of dexa on Glo-I and RAGE staining intensity. After 48 h, dexa significantly increased protein levels of Glo-I and RAGE, as indicated by Western blot (**B1**). Quantifications (**B2**,**B3**) were calculated of at least three independent experiments and are expressed as mean ± SD. * *p* < 0.05. Scale bars: 100 µm. Dexa: dexamethasone, DAB: Diaminobenzidin.

DAB staining in non-stimulated cells indicated a ubiquitous cytosolic Glo-I protein expression and mainly a membrane-related expression of RAGE (Figure 2A). Stimulation of AR42J with dexa, that is required to induce an acinar-like phenotype [38], resulted in an upregulation of Glo-I and RAGE protein expression (Figure 2(B1)). Glo-I expression was increased 1.6 ± 0.3-fold (*p* = 0.03, Figure 2(B2)) and RAGE expression 2.7 ± 0.8-fold (*p* = 0.04, Figure 2(B3)).

### 3.3. CN Induced Glo-I, RAGE, NF-κB and TNF-α

**Figure 3:**
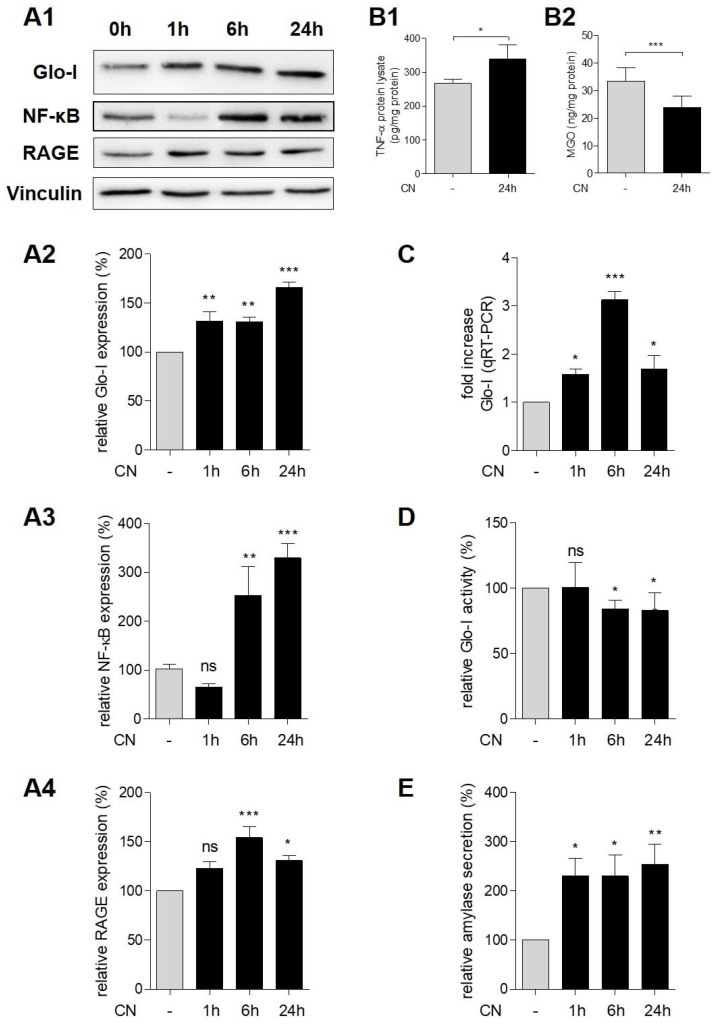
Effect of CN on inflammatory markers and amylase in AR42J. (**A1**–**A4**) Western blot analysis of CN-induced stimulation of Glo-I, NF-κB and RAGE. Representative images indicate time-dependent stimulation of Glo-I, NF-κB and RAGE. Vinculin was used as a housekeeping marker (**A1**). Quantifications of Glo-I (**A2**), NF-κB (**A3**) and RAGE (**A4**) showed statistically significant stimulation after CN treatment. (**B1**,**B2**) ELISA measurements of TNF-α (**B1**) and MGO (**B2**). CN treatment resulted in a significant increase of TNF-α and a decrease of MGO. (**C**) qRT-PCR measurements confirmed Western blot results of Glo-I stimulation upon CN. (**D**) Enzyme kinetic measurement showed a reduction in specific Glo-I activity. (**E**) CN treatment resulted in a time-dependent increase of amylase secretion to supernatant of AR42J cells. Controls received dexa only. Treatment group received dexa with CN. Quantifications were calculated of at least three independent experiments and are expressed as mean ± SD. * *p* < 0.05, ** *p* < 0.01, *** *p* < 0.001. CN: cerulein, Dexa: dexamethasone, RAGE: receptor of advanced glycation end-products, Glo-I: glyoxalase-I, MGO: methylglyoxal.

Incubation of AR42J with CN after pretreatment with dexa increased the protein expression of Glo-I and RAGE in a time-dependent manner (Figure 3(A1,A2,A4)). Glo-I expression increased by up to 166.0% ± 5.6% after 24 h of CN treatment (*p* < 0.001, Figure 3(A2)) and protein levels of RAGE by up to 154% ± 11% after 6 h (*p* < 0.001, Figure 3(A4)). In addition, qRT-PCR measurements indicated a significant increase in Glo-I mRNA transcription that was highest after 6 h of CN-incubation (3.1 ± 0.2-fold increase, *p* < 0.001, Figure 3C). Consecutively, CN treatment led to a slightly but significantly reduced specific Glo-I activity (83.1% ± 13.7% after 24 h compared to controls (100%), *p* < 0.05, Figure 3D). In addition, levels of MGO in protein lysates were significantly decreased upon CN stimulation (23.9 ± 3.9 vs. 33.4 ± 4.9 ng/mg, *p* < 0.001, Figure 3(B2)). Control cells received dexa only for the same incubation period.

CN treatment also resulted in an elevation of pro-inflammatory markers. Incubation of AR42J cells with CN led to a time-dependent increase of NF-κB (p65). Protein expression of NF-κB increased by up to 329.6% ± 29.7% compared to controls (100%, *p* < 0.001, Figure 3(A1,A3)). Furthermore, CN resulted in the stimulation of TNF-α of protein lysates after 24 h of incubation (339.8 ± 40.5 pg/mg compared to 267.3 ± 12 pg/mg, *p* = 0.03, Figure 3(B1)). In addition, CN treatment resulted in a significant increase of amylase secretion of AR42J cells. After 24 h of treatment, amylase levels increased by up to 254.6% ± 39.8% compared to controls (100%, *p* < 0.01, Figure 3E).

### 3.4. Pharmacological Modulation of Glo-I Attenuated CN-Induced Expression of Pro-Inflammatory Markers

**Figure 4:**
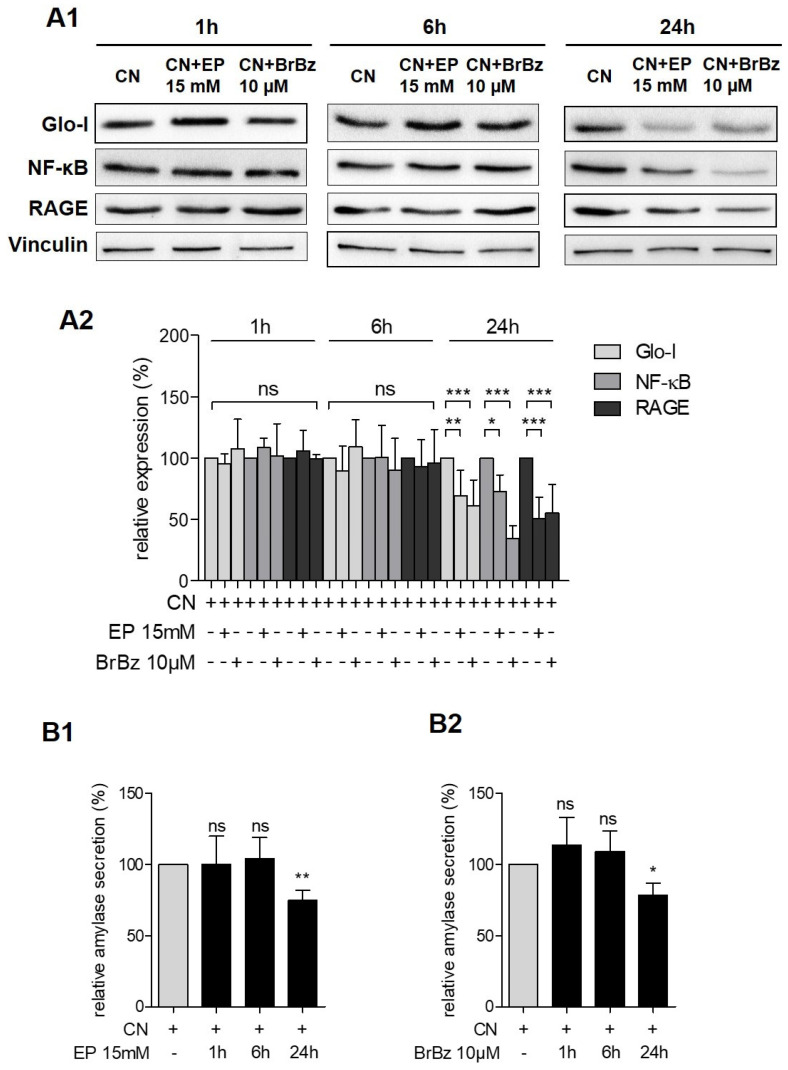
Effect of pharmacological Glo-I modulation on CN treatment. (**A1**,**A2**) Effects of EP and BrBz on CN-induced expression of Glo-I, NF-κB and RAGE. AR42J cells were pretreated with dexa for 48 h. Another treatment with dexa and 15 mM of EP or 10 µM of BrBz with CN showed no effects after 1 or 6 h of incubation. After 24 h, both EP and BrBz significantly reduced the expression of Glo-I, NF-κB and RAGE. Representative images are shown in (**A1**) and quantifications in (**A2**). (**B1,B2**) Impact of co-treatment of EP or BrBz and CN on amylase secretion of AR42J cells. Incubation for 1 and 6 h revealed no significant differences. Co-treatment of 24 h resulted in both EP-CN (**B1**) and BrBz-CN (**B2**) co-treatment in a significantly reduced amylase secretion. Quantifications were calculated of at least three independent experiments and are expressed as mean ± SD. * *p* < 0.05, ** *p* < 0.01, *** *p* < 0.001. EP: ethyl pyruvate, BrBz: S-p-bromobenzylglutathione cyclopentyl diester, CN: cerulein, dexa: dexamethasone, Glo-I: glyoxalase-I, RAGE: receptor of advanced glycation end-products.

The Glo-I modulators EP and BrBz were used as previously described [22,23,24]. AR42J cells were pretreated with dexa for 48 h. Then, additional treatment with dexa ± CN and EP or BrBz was performed. EP or BrBz without CN stimulation resulted in a significant reduction of specific Glo-I activity (Supplementary Figure S2B) but did not influence the expression of Glo-I, NF-κB or RAGE (Supplementary Figure S2(A1,A2,C1,C2)). In contrast, EP and BrBz led to an abrogation of the CN-induced increase in expression of Glo-I, NF-κB and RAGE. Whereas co-treatment of EP or BrBz and CN showed no significant alterations in the referred markers or in amylase secretion after 1 or 6 h, a significant reduction was registered after 24 h of incubation. Treatment with 15 mM of EP and CN resulted in a significant reduction of expression of Glo-I (69.6% ± 20.2%, *p* < 0.01), NF-κB (72.6% ± 13.6%, *p* < 0.05) and RAGE (50.8% ± 17.4%, *p* < 0.001) compared to CN only treated cells (100%, all Figure 4(A1,A2)). In addition, co-treatment of BrBz and CN also led to a significant reduction of Glo-I (61.4% ± 20.28%, *p* < 0.001), NF-κB (34.5% ± 10.4%, *p* < 0.001) and RAGE (55.3% ± 22.2%, *p* < 0.001) related to CN incubation (100%, Figure 4(A1,A2)). Furthermore, both 24 h co-treatment of CN and EP or BrBz significantly reduced amylase secretion (EP: 74.8% ± 7.3%, *p* < 0.01; BrBz: 78.7% ± 8.2%, *p* < 0.05) compared to a solely CN treatment (100%, Figure 4(B1,B2)).

### 3.5. Glo-I Overexpression Reduced CN-Induced Increase of NF-κB, RAGE and Amylase

**Figure 5:**
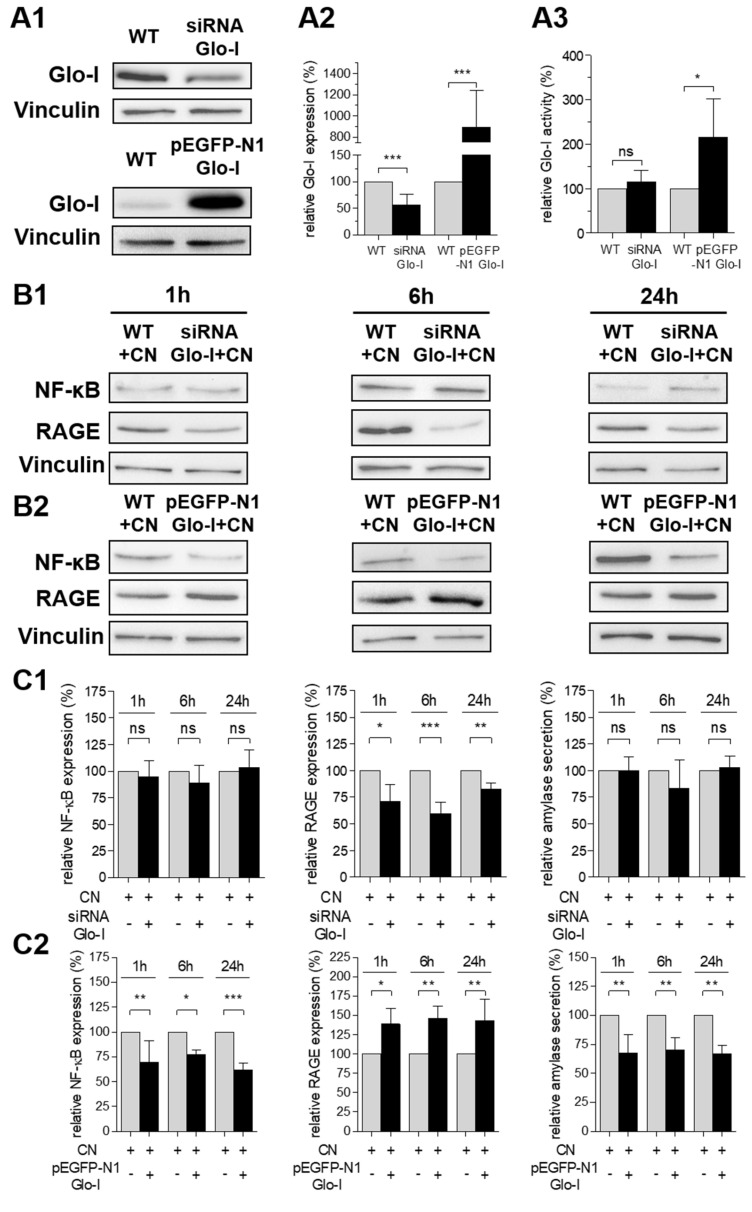
Effect of Glo-I knockdown and overexpression on AR42J. (**A1**–**A3**) Western blot analysis indicated a significant Glo-I knockdown in Glo-I siRNA-treated cells and a significant Glo-I overexpression in pEGFP-N1-Glo-I-treated cells. Representative images are shown in (**A1**), and quantifications in (**A2**). Measurement of specific Glo-I activity (**A3**) revealed significant increases of enzyme activity in Glo-I-overexpressing cells but no alterations in Glo-I siRNA-treated AR42J. (**B1**,**B2**) Representative Western blot images of NF-κB, RAGE and Vinculin (housekeeping) in non-transfected and Glo-I siRNA transfected (**B1**) or pEGFP-N1-Glo-I transfected (**B2**) cells. AR42J were pretreated with dexa and then transfected for 24 h prior to CN stimulation. Cells were incubated for 1, 6 and 24 h with CN. (**C1**,**C2**) Quantifications of B1 (**C1**) and B2 (**C2**) confirmed a significant decrease of RAGE in siRNA-treated cells and a significant increase of RAGE, as well as decrease of NF-κB and amylase secretion in pEGFP-N1-Glo-I transfected cells. Quantifications were calculated of at least three independent experiments and are expressed as mean ± SD. * *p* < 0.05, ** *p* < 0.01, *** *p* < 0.001. CN: cerulein, Glo-I: glyoxalase-I, RAGE: receptor of advanced glycation end-products.

Transfection with Glo-I siRNA or pEGFP-N1-Glo-I vector resulted in a significant decrease (55.8% ± 20.4%, *p* < 0.001) or increase (895.6% ± 347%, *p* < 0.001, Figure 5(A1,A2) and Supplementary Figure S3) of Glo-I expression after 24 h of incubation. Consecutively, the specific Glo-I activity was increased in Glo-I-overexpressing cells (216.0% ± 86.6%, *p* < 0.05) but was not significantly altered in Glo-I siRNA transfected cells (115.4% ± 26.5%, *p* > 0.05, Figure 5(A3)). Note that AR42J cells were pretreated with dexa and then transfected for 24 h prior to CN stimulation. Interestingly, Glo-I siRNA did not influence the expression of NF-κB or amylase secretion after 1 up to 24 h of incubation (*p* > 0.05, Figure 5(B1,C1)) in CN-treated AR42J. In contrast, Glo-I knockdown significantly reduced the CN-induced expression of RAGE (1 h: 71.1% ± 15.6%, *p* < 0.05; 6 h: 59.7% ± 10.5%, *p* < 0.001; 24 h: 82.7% ± 5.5%, *p* < 0.01; Figure 5(B1,C1)). Furthermore, CN treatment of Glo-I-overexpressing AR42J resulted in a significant decrease of NF-κB (1 h: 69.8% ± 21.3%, *p* < 0.01; 6 h: 77.5% ± 4.2%, *p* < 0.05; 24 h: 61.8% ± 6.5%, *p* < 0.001; Figure 5(B2,C2)) and amylase (1 h: 67.6% ± 16.0%, *p* < 0.01; 6 h: 70.3% ± 10.3%, *p* < 0.01; 24 h: 66.6% ± 7.4%, *p* < 0.01; Figure 5(B2,C2)) compared to CN treatment of non-transfected cells (100%). Conversely, CN incubation of Glo-I-overexpressing cells led to an increase of RAGE expression (1 h: 138.6% ± 20.2%, *p* < 0.05; 6 h: 146.0% ± 15.0%, *p* < 0.01: 24 h: 143.3% ± 27.5%, *p* < 0.01; Figure 5(B2,C2)) related to CN treatment of non-transfected cells (100%).

### 3.6. Effects of Pharmacological Modulation and Overexpression of Glo-I Are Mediated via Regulation of MGO

**Figure 6:**
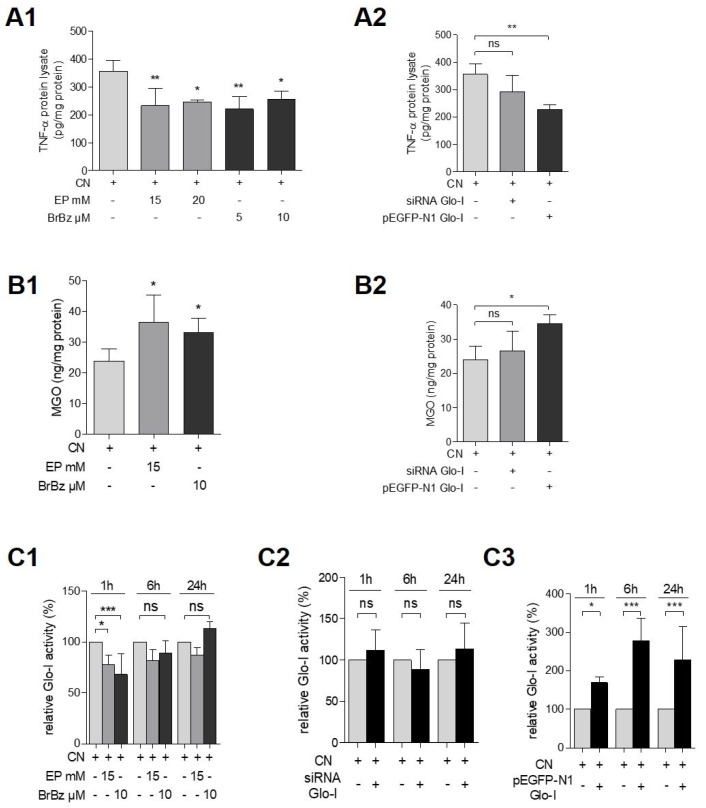
Effect of Glo-I targeting on TNF-α and MGO. (**A1**,**A2**) Effect of pharmacological modulation and Glo-I knockdown or overexpression on TNF-α levels of CN-treated AR42J protein lysates. EP, BrBz (**A1**) and Glo-I overexpression (**A2**) significantly decreased CN-induced TNF-α concentrations. Glo-I knockdown did not statistically significantly reduce TNF-α (**A2**). (**B1**,**B2**) Effect of EP, BrBz, Glo-I knockdown and overexpression on MGO levels of CN-treated cells. EP, BrBz (**B1**) and Glo-I overexpression (**B2**) increased MGO compared to CN incubation and thus restored MGO to levels of untreated cells (see Figure 3). GLo-I knockdown revealed no significant impact on MGO (**B2**). (**C1**–**C3**) Analysis of specific Glo-I activity after co-treatment of CN and EP or BrBz, as well as Glo-I overexpression or knockdown. EP and BrBz significantly reduced Glo-I activity (**C1**) in CN-treated cells but Glo-I overexpression (**C3**) resulted in an increase of Glo-I activity. Glo-I siRNA (**C2**) did not influence Glo-I activity in CN-treated AR42J. Quantifications were calculated of at least three independent experiments and are expressed as mean ± SD. * *p* < 0.05, ** *p* < 0.01, *** *p* < 0.001. EP: ethyl pyruvate, BrBz: S-p-bromobenzylglutathione cyclopentyl diester, CN: cerulein, Glo-I: glyoxalase-I, MGO: methylglyoxal.

AR42J incubated for 24 h with EP and CN resulted in a significant decrease (15 mM: 232.7 ± 61.0 pg/ng, *p* < 0.01; 20 mM: 246.3 ± 5.7 pg/ng, *p* < 0.05) of CN-induced TNF-α stimulation (355.5 ± 39.8 pg/ng). These results were confirmed by a second Glo-I modulator, BrBz (5 µM: 220.4 ± 44.8 pg/ng, *p* < 0.01; 10 µM: 255.0 ± 30.6 pg/ng; Figure 6(A1)). In addition, EP (15 mM: 36.56 ± 8.8 ng/mg; *p* < 0.05) and BrBz (10 µM: 33.3 ± 4.6 ng/mg; *p* < 0.05) resulted in abrogation of the CN-induced reduction of MGO (23.9 ± 3.9 ng/mg; Figure 6(B1)). This restoration of MGO levels was accompanied by a further decrease in specific Glo-I activity (EP 15 mM: 77.9% ± 9.1%, *p* < 0.05; BrBz 10 µM: 67.9% ± 20.5%, *p* < 0.001; Figure 6(C1)) after 1 h of incubation. This decrease was also detectable after 6 and 24 h but remained statistically insignificant. In addition, Glo-I overexpression also significantly reduced the levels of CN-induced TNF-α (228.7 ± 16.5 pg/mg, *p* < 0.01, Figure 6(A2)) and also abrogated the CN-induced reduction of MGO (34.5 ± 2.5 ng/mg, *p* < 0.05, Figure 6(B2)). In addition, Glo-I overexpression increased specific Glo-I activity (24 h: 277.7% ± 59%, *p* < 0.001, Figure 6(C3)) compared to unique CN treatment (100%). In contrast, CN treatment of Glo-I siRNA transfected AR42J did not significantly influence TNF-α levels, MGO or Glo-I activity (Figure 6(A2,B2,C2)) compared to CN-treated non-transfected cells.

## 4. Discussion

ROS are important species in the development of AP [2]. Amongst others, ROS can be formed by the modification of proteins, nucleic acids and lipids with the highly reactive compound MGO [39]. Therefore, an adequate detoxification of MGO by the major detoxifying enzyme, Glo-I, remains essential to maintain cell viability and functionality [40]. Nevertheless, the impact of Glo-I in acute pancreatitis has not been elucidated so far.

In our study, we showed that Glo-I seems to be important during the development and differentiation of pancreatic acinar cells. As Glo-I is ubiquitously expressed in all mammalian cells [41], we also found a high expression in AR42J cells. Moreover, during differentiation to an acinar-like phenotype, that was induced by dexamethasone [38], the expression of Glo-I and RAGE significantly increased. Compared to our findings, data suggest that Glo-I expression depends on differentiation and proliferation of cells. This indicates that Glo-I expression may vary based on cell subtype and functionality [20].

Furthermore, our data clearly highlighted that Glo-I is overexpressed in CN-induced AP, in vitro and in vivo. This increase in Glo-I expression was accompanied by a CN-mediated induction of pro-inflammatory markers, such as NF-κB, RAGE and TNF-α. Moreover, targeting Glo-I by two independent pharmacological modulators, EP and BrBz, as well as overexpression or silencing of Glo-I by siRNA, influenced the CN-induced production of inflammatory markers. EP and BrBz significantly reduced the CN-stimulated expression of Glo-I, NF-κB, RAGE, TNF-α and amylase secretion. Consecutively, Glo-I overexpression significantly ameliorated the induction of NF-κB, TNF-α and secretion of amylase upon CN treatment. On the contrary, Glo-I silencing could only impact on RAGE expression and did not show any significant effect on NF-κB, TNF-α or amylase secretion. This missing influence of Glo-I silencing could be related to the limited effect of the siRNA-mediated reduction in Glo-I expression (to about 50%) compared to the distinct stimulation of Glo-I overexpression after transfection (about a 9-fold increase). Moreover, further minor detoxifying enzymes, e.g., the aldose reductase or the glyoxalase-III [42], are capable to prevent, at least temporarily, toxic levels of MGO in case of Glo-I dysfunction [43]. This fact could also explain the observation of a missing increase in MGO levels that would have been suggested by Glo-I knockdown. In addition, specific Glo-I activity remained unchanged, although protein production was significantly reduced in Glo-I-silenced cells. Thus, a reduction in Glo-I expression was accompanied by an increase in the enzymatic activity of the remaining proteins, which further supports our assumption.

As a consequence of the aforementioned results, the contribution of Glo-I to the CN-induced AP appears to be considerable. Nevertheless, some results may seem somewhat counterintuitive and need further explanation. First, our data clearly show reduced MGO levels upon CN treatment, although several data indicate an increase in MGO in inflammatory diseases [11,22,23,44]. On the other hand, this reduction of MGO in CN-treated cells is comprehensible given the CN-mediated induction of Glo-I expression and the slightly reduced specific activity of Glo-I upon CN treatment. Altogether, the effects of CN on Glo-I expression, activity and MGO levels are shown, but it still remains unclear if the stimulation of Glo-I is a cause or a consequence of altered MGO levels. Thus, one could argue that an increased expression of Glo-I indicates a protective anti-inflammatory mechanism in AP.

Our results are further supported by the data of the two Glo-I modulators. Both EP and BrBz resulted in a reduction of specific Glo-I activity as well as a reduction in Glo-I expression, but only in CN-treated cells. This deterioration of Glo-I seems disadvantageous as it was accompanied by a consecutive slight but significant increase in MGO. Nevertheless, the resulting regulation of MGO at sub-toxic levels could explain the anti-inflammatory effects of both EP and BrBz. While MGO is toxic at high concentrations, a low level of MGO has been shown to activate transcription factors [45] and modify proteins [46]. In this regard, different MGO levels could lead to temporary activation or inhibition of biochemical targets that are regulated via Glo-I activity. Our results in the reduction of Glo-I, followed by an increase of MGO upon EP or BrBz, further support these findings. Indeed, previous studies suggest that MGO could act as an intracellular mediator of the action of Glo-I inhibitors, which is in line with our results [47]. Recent data also show an inhibitory effect of MGO on NF-κB [48] that could be explained by the transcriptional control of Glo-I by Nrf2 in response to MGO [49,50]. All of these findings highlight the complex regulation of Glo-I and MGO, however they also provide evidence for an essential role of this enzymatic system for AP. Thus, EP and BrBz could regulate the MGO levels by (partial) inhibition of Glo-I and therefore induce the presented effects.

Furthermore, our results regarding the expression of RAGE need further explication. A pharmacological modulation of Glo-I by EP or BrBzGSHCP_2_ resulted in a significant reduction of the CN-induced increase of RAGE. On the other hand, Glo-I silencing by siRNA reduced and Glo-I overexpression induced the expression of RAGE, which are conflicting results. However, recent data reveal that there is a feed-forward loop in AGE production, RAGE activation and receptor-mediated propagation of ongoing AGE formation and accumulation in the tissues through downregulation of counter-regulatory mechanisms [51]. In detail, the authors found a downregulation of Glo-I as a consequence of RAGE activation that indicates a vicious cycle of inflammation. In relation to our results, the induced overexpression or knockdown of Glo-I by means of siRNA or plasmid transfection affects this feed-forward loop. The induced Glo-I overexpression resulted in an increase of RAGE as a counter-regulatory mechanism. Additionally, knockdown of Glo-I would lead to the opposite result.

In addition, our study has some limitations. First, we analyzed Glo-I in a mouse model of CN-induced AP but did not investigate further models, e.g., the bile-duct ligation model. However, CN-induced AP has been known for decades and an established and reliable model to study AP has been demonstrated [27]. Second, we did not evaluate the effects of a pharmacological treatment in wild-type mice nor analyze the effects of CN-induced AP in Glo-I knockout mice. As these mice are very recently available [52], these experiments are foreseen in future projects. Nevertheless, our study provides robust data for the involvement of Glo-I in AP, and thus represents important basics for further research in this scientific field. In addition, our results were strengthened by analyzing different housekeeping markers and the influence of CN treatment on them. We could clearly show that β-actin and GAPDH, although often used in related publications, are strongly influenced by CN and should therefore not be used in models of CN-induced AP. Our data indicated PPIB as a robust housekeeping gene for qRT-PCR analysis and Vinculin for Western blot. Therefore, these markers should be the preferred housekeeping markers in an experimental setting investigating the effects of CN.

## 5. Conclusions

In conclusion, our data showed an overexpression of Glo-I in a model of CN-induced AP. A pharmacological modulation and overexpression of Glo-I reduced the secretion of amylase and the release of pro-inflammatory cytokines. Therefore, targeting Glo-I in AP demonstrated an interesting approach for future in vivo studies of AP.

## Data Availability

The data presented in this study are available in article and supplementary material.

## Supplementary Materials

The following are available online at https://www.mdpi.com/article/10.3390/antiox10101574/s1, Figure S1: Effect of CN on severity of pancreatitis in mice, Figure S2: Effects of EP and BrBz on Glo-I, NF-κB and RAGE, Figure S3: Glo-I overexpression and knockdown in AR42J, Figure S4: Effect of CN on housekeeping markers.

## Abbreviations

AGE	advanced glycation end-products
AB	antibody
AP	acute pancreatitis
bw	body weight
BrBz	BrBzGSHCP_2_ (S-p-bromobenzylglutathione cyclopentyl diester)
CN	cerulein
DAB	Diaminobenzidin
DAMP	damage-associated molecular pattern
Dexa	dexamethasone
EP	ethyl pyruvate
FCS	fetal calf serum
Glo-I	Glyoxalase-I
Glo-II	Glyoxalase-II
GSH	L-gluthatione
MGO	methylglyoxal
NLRP3	nucleotide-binding oligomerization domain-like receptor protein 3
P/S	penicillin and streptomycin
PVDF	polyvinylidene fluoride
qRT-PCR	quantitative real-time polymerase chain reaction
RAGE	receptor for advanced glycation end-products
ROS	oxidative stress
SDS-PAGE	sodium dodecyl sulfate polyacrylamide gel electrophoresis
