# Supplementary material for: Glyoxalase-I Is Upregulated in Acute Cerulein-Induced Pancreatitis: A New Mechanism in Pancreatic Inflammation?
Source: Antioxidants (Basel). 2021 Oct 5;10(10):1574. doi: 10.3390/antiox10101574 (PMC8533479; doi:10.3390/antiox10101574)
Supplement: Supplementary file 1 [file antioxidants-10-01574-s001.zip › antioxidants-1275795-supplementary.pdf]

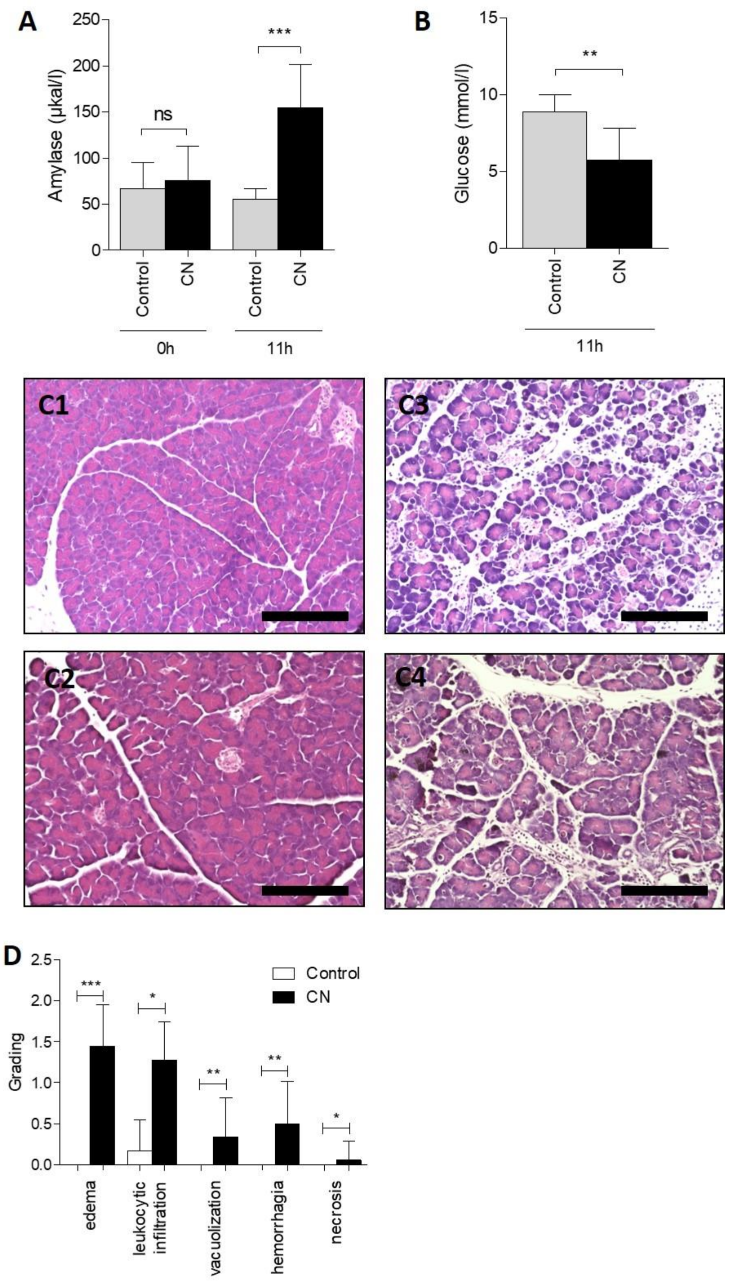

Supplemental Figure 1: Effect of CN on severity of pancreatitis in mice. A-B, Effect of CN on amylase levels and glucose in mice. CN-treated mice revealed a significant increase of amylase levels (up to 3 fold) compared to controls **(A)**. In addition, CN treatment significantly reduced glucose levels **(B)**. Quantification were calculated of 16 animals (8 per group) and are expressed as mean ± S.D. **C1-C4**, representative images of HE-staining of CN-treated **(C3-C4)** and control animals **(C1-C2)**. Note the edema and infiltration of inflammatory cells in the CN group. **D**, quatifications of grading of severity of pancreatitis. Six slides per pancreas were analyzed for grading of edema (0 = no; 1= interlobular, 2 = + moderate intralobular, 3 = + severe intralobular edema), leukocytic infiltration (0 = absent, 1 = scarce perivascular, 2 = moderate perivascular and scarce diffuse, 3 = abundant diffuse infiltration), vacuolization (0 = absent, 1 = < 25%, 2 = 25-50%, 3 = > 50% of acinar cells), hemorrhagia (0 = no, 1 = 1-2, 2 = 3-5, 3 = > 5 hemorrhagic foci per slide), and necrosis (0 = no, 1 = < 15 %, 2 = 15-35 %, 3 = > 35 % of cells). Results are expressed as mean ± S.D. from 8 animals (4 male, 4 female) per group. Scale bars: 100 µm. * $P<0.05$, ** $P<0.01$, *** $P<0.001$. CN: cerulein. HE: hematoxylin eosion. Note that international SI-units were used (µkat/l = U/l x 0.0167).

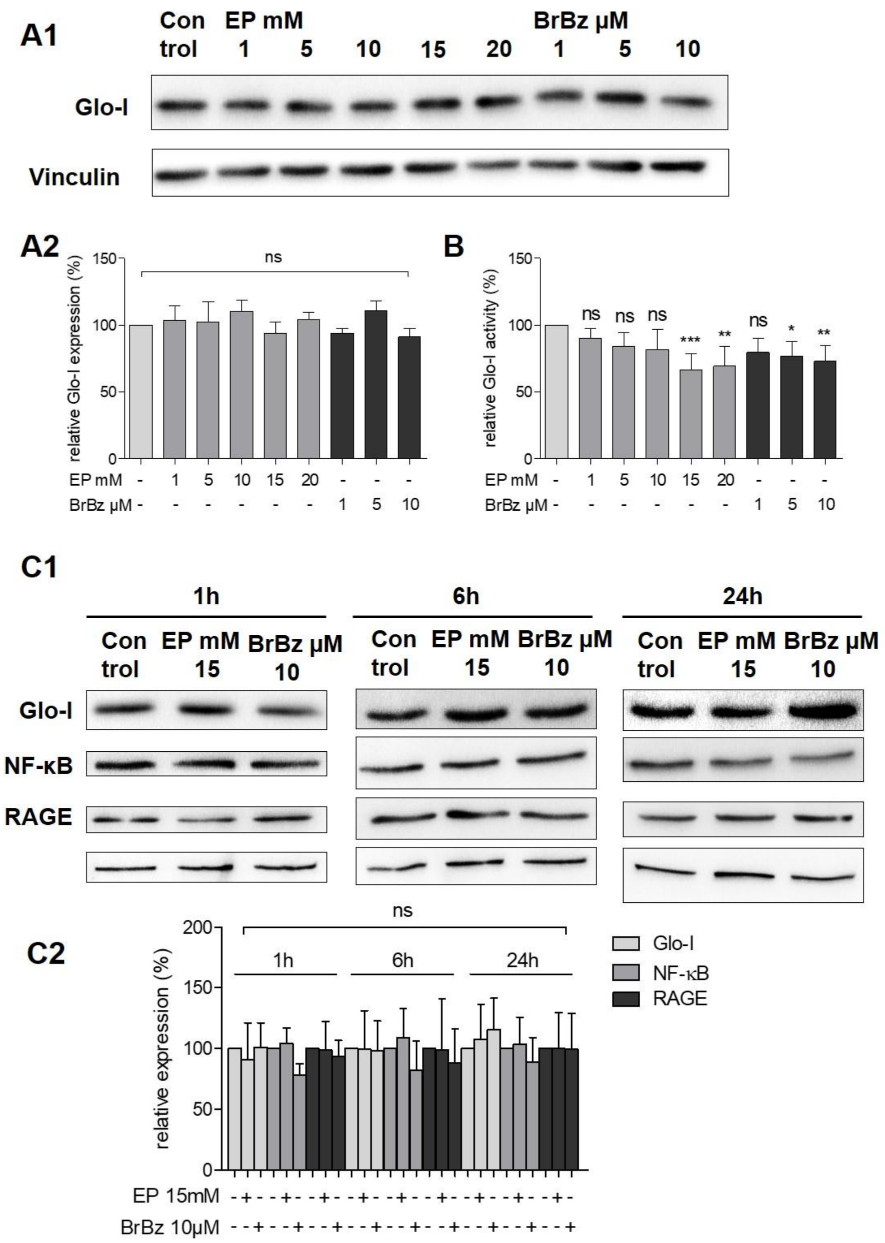

Supplemental Figure 2: Effects of EP and BrBz on Glo-I, NF-κB and RAGE. A1-A2, Effect of different doses of EP and BrBz on Glo-I expression. Neither EP nor BrBz significantly influenced protein expression of Glo-I in AR42J without CN treatment. Representative images are shown in **A1**, quantifications in **A2**. **B**, 15-20 mM EP and 5-10 µM BrBz significantly reduced specific Glo-I activity in AR42J that were not treated with CN. **C1-C2**, Effects of EP and BrBz on Glo-I, NF-κB and RAGE at different incubation times. Neither EP nor BrBz significantly influenced protein expression of Glo-I, NF-κB or RAGE in AR42J without CN treatment after an incubation of 1 up to 24 hours. Representative images are shown in **(C1)**, quantifications in **(C2)**. Quantifications were calculated of at least three independent experiments and are expressed as mean ± S.D. *P<0.05, **P<0.01, ***P<0.001. EP: ethyl pyruvate, BrBz: S-p-bromobenzylglutathione cyclopentyl diester, CN: cerulein, Glo-I: Glyoxalase-I, RAGE: receptor of advanced glycation endproducts.

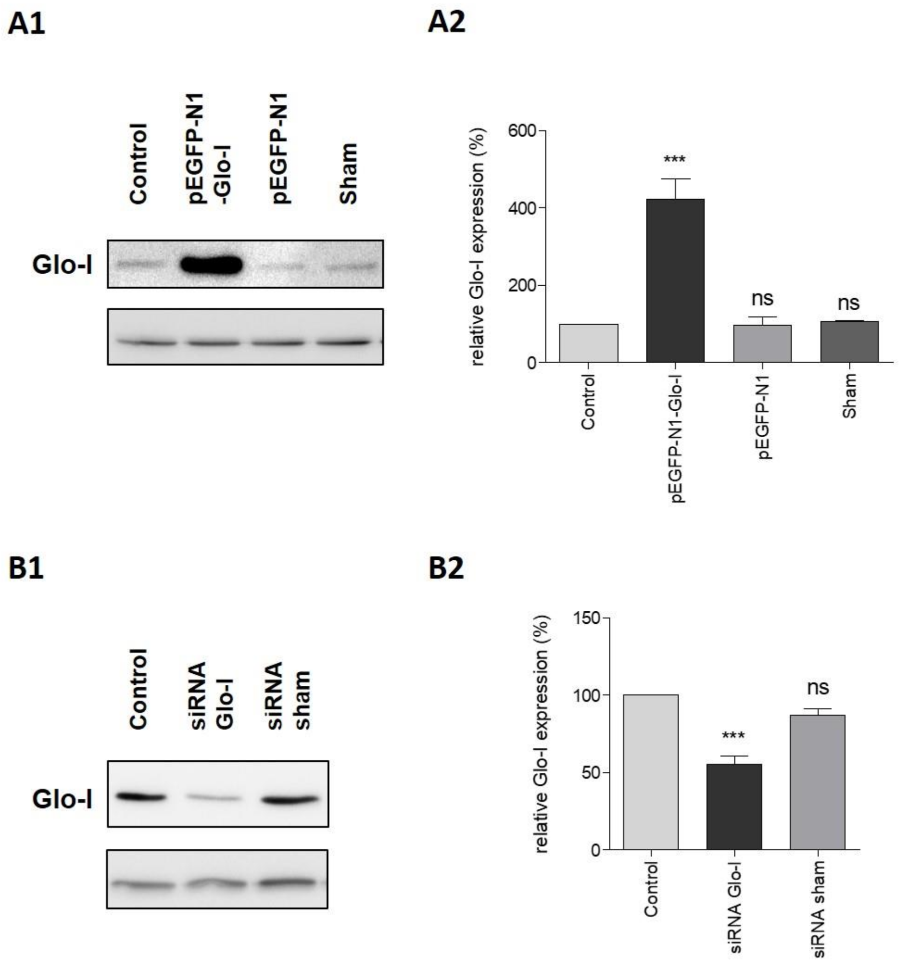

Supplemental Figure 3: Glo-I overexpression and knockdown in AR42J. A1-A2, Glo-I overexpression. AR42J were transfected for 24 hours with the pEGFP-Glo-I vector. Representative Western Blot images **(A1)** showed an upregulation of Glo-I, whereas control vector and sham transfected cells indicated no increase in Glo-I expression. Quantifications **(A2)** confirmed a significant upregulation of Glo-I. **B1-B2**, Glo-I knockdown. AR42J were transfected for 24 hours with Glo-I siRNA. Western Blot images **(B1)** showed a decrease in Glo-I expression in Glo-I siRNA tranfected cells but not in sham transfected cells. Quantifications **(B2)** revealed a significant reduction in Glo-I expression up on Glo-I siRNA treatment. Quantifications were calculated of at least three independent experiments and are expressed as mean ± S.D. ***P<0.001. Glo-I: Glyoxalase-I.

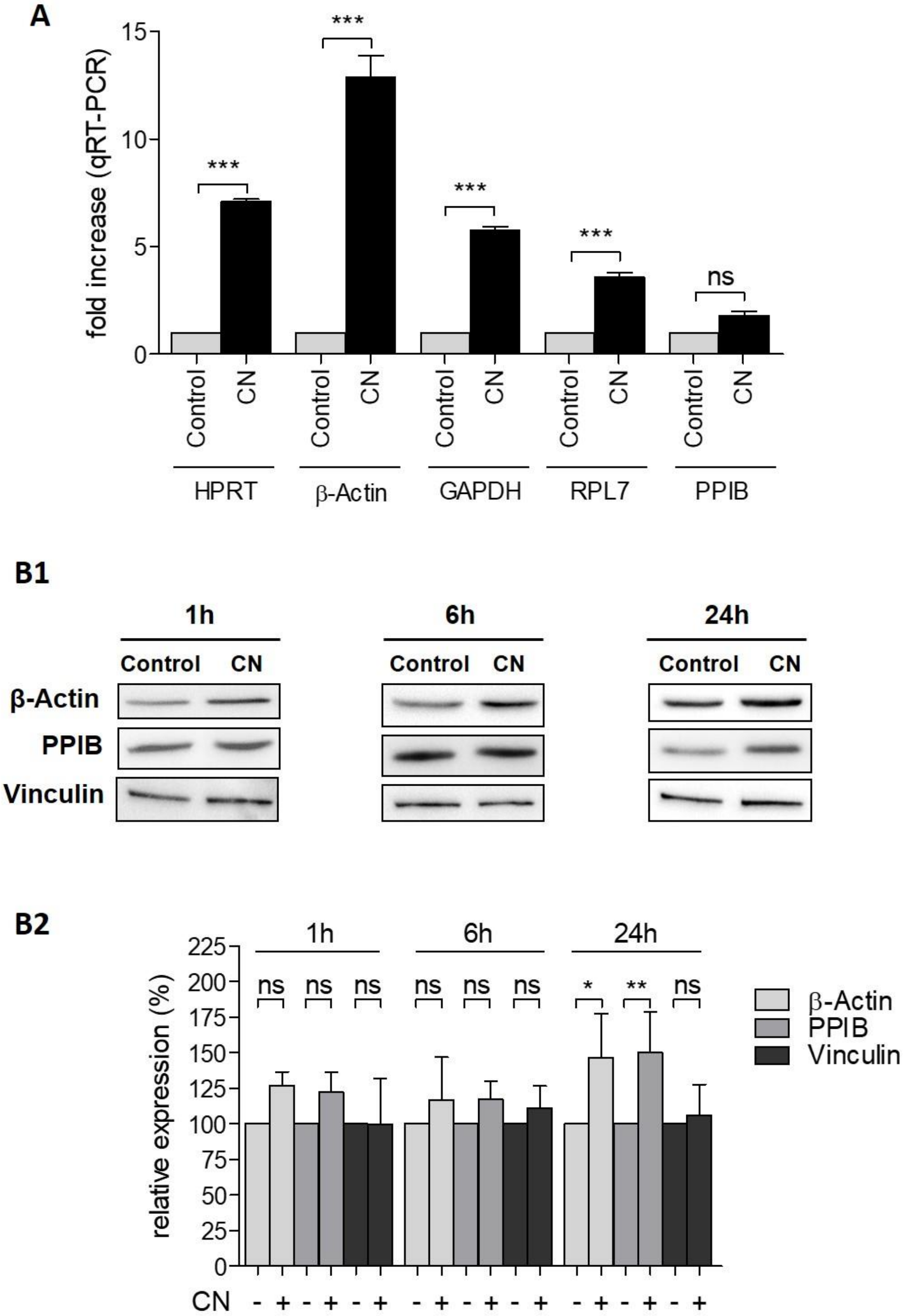

Supplemental Figure 4: Effect of CN on housekeeping markers. A, Analysis of CN influence on qRT-PCR housekeeping genes. Data indicated that only PPIB was not significantly upregulated by CN. All other housekeeping markers were significantly influenced. **B1-B2**, Effect of CN on housekeeping markers. Representative images are shown in **B1**. Quantifications **(B2)** showed that only Vinculin was not significantly induced by CN after 24 hours of incubation. For incubation times of 1 hour and 6 hours, no significant effect of CN on β-Actin, PPIB or Vinculin was demonstrated. Quantifications were calculated of at least three independent experiments and are expressed as mean ± S.D. *$P<0.05$, **$P<0.01$, ***$P<0.001$. CN: cerulein.
